# Managing incursions of *Vespa velutina nigrithorax* in the UK: an emerging threat to apiculture

**DOI:** 10.1038/s41598-020-76690-2

**Published:** 2020-11-11

**Authors:** Eleanor P. Jones, Chris Conyers, Victoria Tomkies, Nigel Semmence, David Fouracre, Maureen Wakefield, Kirsty Stainton

**Affiliations:** 1 Fera Science Ltd, York Biotech Campus, Sand Hutton, York, YO41 1LZ UK; 2 Animal and Plant Health Agency, York Biotech Campus, Sand Hutton, York, YO41 1LZ UK; 3 The Pirbright Institute, Ash Road, Pirbright, Woking, GU24 0NF UK

**Keywords:** Ecology, Zoology

## Abstract

*Vespa velutina nigrithorax* is an invasive species of hornet accidentally introduced into Europe in 2004. It feeds on invertebrates, including honey bees, and represents a threat to European apiculture. In 2016, the first nest of this hornet was detected and destroyed on mainland UK. A further 8 nests were discovered between 2016 and 2019. Nest dissection was performed on all nests together with microsatellite analyses of different life stages found in the nests to address the reproductive output and success of nests found in the UK. None of the nests had produced the next generation of queens. Follow-up monitoring in those regions detected no new nests in the following years. Diploid males were found in many UK nests, while microsatellite analysis showed that nests had low genetic diversity and the majority of queens had mated with one or two males. All UK nests derived from the European zone of secondary colonisation, rather than from the native range of the species. None of the nests discovered so far have been direct offspring of another UK nest. The evidence suggests that these nests were separate incursions from a continental population rather than belonging to a single established UK population of this pest.

## Introduction

The yellow-legged Asian hornet (*Vespa velutina nigrithorax*) is an invasive species of hornet which presents a threat to invertebrate populations, in particular to honey bees which constitute a large proportion of the hornet’s diet^1^. *Vespa velutina nigrithorax* is extremely successful at colonising new areas; the hornet spread through France, where it was accidentally introduced around 2004, at a rate of expansion of between 75 and 82 km/year^2^ while an incursion of the hornet into Korea spread at 12.4 km/year^3^. *Vespa velutina nigrithorax* has gone on to colonise Spain in 2010, Portugal and Belgium in 2011, Italy in 2012^4,5^ and Germany in 2014^6^. It now inhabits approximately three quarters of France and has become established on the Channel Islands^7^ (which are not part of the UK).

A single *V. velutina nigrithorax* queen can produce thousands of individuals, on average 6000, subject to a sufficiently protein rich diet^1^. A single mated queen can travel over 40 km per day, quickly colonising new regions and producing thousands of individuals during the summer months^8^. The founding queen will produce male and then female offspring before the end of the season. The females from this final brood (referred to as gynes) will become the founding queens of the next generation and go on to disperse and colonise new areas^8^. In France, a single colony will produce up to 350 gynes between September and November^1^.

Asian honey bees, *Apis cerana*, have various defence strategies against invading hornets including intimidation behaviours (abdominal shaking, emitting an alarm sound), forming physical barriers (i.e. bee carpets or building walls of propolis) and “bee-balling”^9^. The defensive response in European honey bees, *Apis mellifera*, is insufficient to fend off hornets and leads to a loss of workers due to direct predation as well as decreased foraging activity, resulting in a progressive weakening of the colony. In France, the hornets are reported to intensively forage on honey bee colonies and estimates from losses in France suggest hornets can cause 20–30% colony loss^7^. *Vespa velutina nigrithorax* represents yet another serious threat to honey bees, and other pollinators, in Europe.

In 2016, the first hornet nest in England was discovered in Tetbury^10^ and subsequently nests have been discovered in geographically dispersed locations. Each nest was euthanized by the Animal and Plant Health Agency and sent to Fera Science Ltd for analysis. The priorities for nest removal were operator safety and to ensure the nest was rendered inviable, and adult hornets are likely to have been lost from the nests during this process. Nests were dissected and morphologically analysed and individuals of each life stage taken for microsatellite analysis. This study reports on the relatedness of the mainland UK hornets and the reproductive status of the nests.

## Materials and methods

### Samples

**Figure 1:**
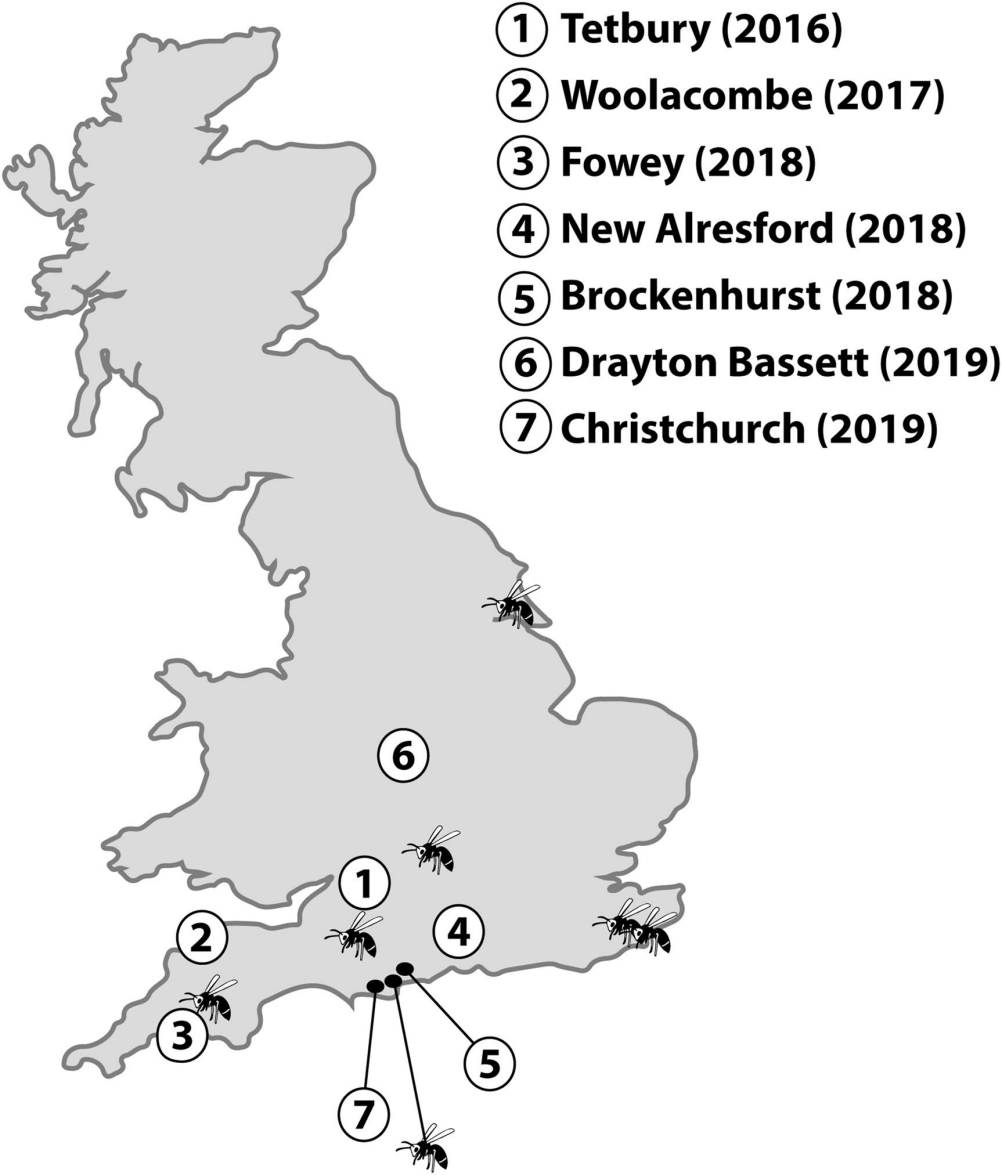
Map of locations of nests of *Vespa velutina nigrithorax* discovered in the UK. These were: (1) one nest from Tetbury, Gloucestershire (2016), (2) one nest from Woolacombe, Devon (2017), (3) two nests from Fowey, Cornwall (2018), (4) one nest from New Alresford, Hampshire (2018), (5) one nest from Brockenhurst, Hampshire (2018), (6) one nest from Drayton Bassett, Staffordshire (2019) and (7) two nests from Christchurch, Dorset (2019). The hornet icon denotes the locations of individual hornets found.

All Asian hornet nests found in the UK (all from England) to the end of 2019 were included in the study (Fig. 1). Lone adult individuals were caught away from the nest at these locations, and additional individuals were recovered in 2016 from Somerset (adult female, sample too degraded to identify if it was a worker or queen), in 2018 from Hull (adult female worker), from Oxfordshire (adult worker), from Liskeard in Cornwall (diploid male) and from Dungeness, Kent (2 adult males), and in 2019 in New Milton in Hampshire (adult female worker) and in Tenterden in Kent (adult female worker).

### Nest collection and dissection

The primary focus for the nest destruction is eradication of the colony. It is inevitable that there will be some loss of adult hornets during destruction and removal of the nest. However, APHA took steps to minimise the loss of hornets and unnecessary damage to the remaining nest. Nests were detected through field observations, and located and removed by APHA wildlife officers, trained in Asian hornet nest destruction. Nests were destroyed close to dusk which enables the team to operate safely and maximises the number of hornets in the nest as they are diurnal. Nests were left overnight for the pesticide to take effect, before removal the following morning when nests were sealed in two biohazard bags and frozen. Any hornets remaining in the vicinity, underneath nests or in traps, were also collected. Nests were frozen after collection for a minimum of 72 h. The loss of hornets during the destruction process are unlikely to adversely impacted the genetic analysis.

The nests were examined and the number of adult hornets, sex ratio and mass of individuals were recorded. Individual adult hornet wet weights were obtained using an analytical balance (Sartorius R2000; ± 0.1 mg). The diameter of the nest and each individual comb was measured; the size of the largest comb is indicative of the number of individuals produced by the nest^8^. The life stages present in the nest were determined. Teneral adults were determined as fully formed adults that were in sealed cells prior to exoskeleton hardening and final colouration.

### DNA sampling

Tissue samples were taken from selected individuals for DNA analysis. For each nest, samples were taken from at least ten individuals of each life stage present (adults, teneral adults, pupae, larvae and eggs). All adults found away from a nest were sampled. Single hind legs were removed from adults, teneral adults and pupae, while for larvae a section was removed (avoiding the gut), all using single-use scalpel blades. Eggs were sampled whole. Leg tissue was cut into small sections and disrupted with a micro-pestle. DNA was extracted from all samples using the DNeasy blood and tissue kit (QIAGEN) following the manufacturer’s protocol.

### Microsatellite marker analysis

Samples were genotyped for fifteen microsatellite markers (Arca et al. 2015)^11^: R1-36, LIST2020, R1-80, R4-33, R4-114, R1-169, D2-185, LIST2018, D3-15, VMA6, LIST2015, R4-26, R1-75, R4-100, VMA8 using fluorescent tagged primers (FAM, NED or HEX). They were run in six multiplexed PCR reactions using Multiplex PCR kits (QIAGEN). Primer concentrations were optimised for each multiplex; cycling conditions were 15 min at 95 °C, followed by 35 cycles of 30 s at 94 °C, 90 s at 59 °C, 60 s at 72 °C, and a final elongation step of 30 min at 60 °C. PCR products were genotyped on an ABI 3031 Genetic Analyzer using the ROX 500 ladder as a size standard. Genotypes were scored into allele sizes using Geneious 9.1.4 and error rates estimated from scoring discrepancies between sample repeats. Kinship analyses (inferring maternal and paternal genotypes per nest, assessing whether offspring were full siblings, half siblings or not siblings) were completed using COLONY2^12,13^ and/or inferred manually. Ploidy was inferred from marker zygosity; where all markers were homozygous, it was assumed the sample was a haploid. Data were compared to microsatellite results from^11^ using samples from that study to ‘calibrate’ allele sizes on the different machines. Allele frequencies per location were calculated in CONVERT^14^ Genetic diversity indices (allelic diversity, observed and unbiased expected heterozygosity) were calculated per nest in Genetix^15^, excluding haploids.

Genotypes from lone individuals were assessed, considering whether they derived from a recovered nest of that year (i.e. their genotype was compatible with being an offspring of the nest parental genotypes) or from an unrecorded nest, primarily by assessing whether the lone individuals had alleles absent from the nest parental genotypes. In addition, Factorial Correspondence Analysis plots of all individuals sampled were run in Genetix^15^ as a second method to visualise whether lone individuals clustered with nest individuals (data not shown).

## Results

### Nests

A summary of the nest results can be found in Tables 1 (physical description of nests) and 2 (ploidy). All adult females that were weighed were classed as workers or founder queens using the information found in Rome et al.^8^, which defined a limit of 593 mg wet weight or 250 mg dry weight to discriminate between workers and founder queens. The average wet weight of founder queens in September was 624 mg (N = 5). All nests recovered had fewer adults present than expected, this was presumed to be due to the loss of adult hornets during the destruction process and subsequent removal of the nest from its original location.

**Table 1:** Summary of observations from all nests discovered in the UK.

Nest date and location	Date destroyed	Nest diameter (cm)	Number of combs	Comb diameters (cm)	Sex of adults (morphological)^a^	Brood present	Haploid egg production (estimated)	N. paternal genotypes (estimated)	N diploid males/genotyped males^ab^
Tetbury	28-Sep-16	23	5	20, 23, 21, 16, 7.5	13♂, 57♀^e^	All stages	21st September	1	9/9
Woolacombe	27-Sep-17	27	7	25, 24, 27, 20, 16, 24, 12	166♀	All stages	02nd September	2–3	n. a
Fowey 1	06-Sep-18	15	3	15, 15, 12	3♂	No eggs or larvae	n.a	1	3/3
Fowey 2	21-Sep-18	19	4	18, 19, 16.5, 12	7♂, 8♀	All stages	30th August	1^c^	10/10
New Alresford	25-Sep-18	18	4	13.5, 18, 17, 14	28♂, 94♀	All stages	03rd September	2	10/10
Brockenhurst	04-Oct-18	18.5	3	18, 18.5, 16	5♂, 13♀	No eggs	29th August	2	0/5
Drayton Bassett	04-Sept-19	n.d.^d^	n.d.^d^	n.d.^d^	5♀	All stages	n.a	1	n. a
Christchurch 1	03-Oct-19	13	2	6, 8	1♀queen	Eggs & early larvae	01 October	1	n. a
Christchurch 2	10-Oct-19	n.d.^d^	2	n.d.^d^	No adults present	No eggs	n.a	1	n. a

^a^Adults were lost from some nests during nest destruction.

^b^Up to 10 individuals identified morphologically as males were genotyped per nest, and could either be haploid (“true” males) or diploid.

^c^The genetic diversity data were calculated for the individuals for Fowey 1 and 2 combined, given they were offspring from a single queen.

^d^No data; nest too damaged.

^e^The symbol ♀ denotes a worker female unless suffixed by “queen”.

**Table 2:** Ploidy of nests.

Nest	Eggs	Larvae	Pupae	Teneral adults	Adults
Tetbury	9 haploid, 2 diploid	20 diploid	20 diploid	20 diploid	22 diploid
Woolacombe	7 haploid, 2 diploid	Failed*	2 haploid, 8 diploid	10 diploid	10 diploid
Fowey 1	n.a	10 diploid	10 diploid	10 diploid	3 diploid
Fowey 2	9 haploid	6 haploid, 4 diploid	10 diploid	10 diploid	10 diploid
Brockenhurst	n.a	9 haploid	10 haploid	10 haploid	9 haploid, 11 diploid
New Alresford	2 haploid, 5 diploid	5 haploid, 5 diploid	10 diploid	10 diploid	10 diploid
Drayton Bassett	10 diploid	8 diploid	7 diploid	9 diploid	6 diploid
Christchurch 1	2 haploid, 8 diploid	10 diploid	n.a	n.a	1 diploid
Christchurch 2	n.a	10 diploid	10 diploid	10 diploid	n.a

n.a. None available. This life stage was not found in the nest.

*Samples failed to amplify.

#### Tetbury

The findings from the examination of the Tetbury nest have been described previously^10^ but are briefly summarised again here. This nest was discovered on 28th September 2016**.** In total, 70 adult hornets were found in the nest. The wet weight of 57 adult female hornets ranged from 202 to 322 mg with a mean of 256 mg (N = 19), whilst that of 13 adult male hornets ranged from 248 to 326 mg with a mean of 290 mg (N = 7). The nest diameter was 23 cm and the nest contained five combs, four of which contained all life stages (eggs, larvae, pupae, teneral adults, adults) of the Asian hornet. All life stages examined were diploid. The nest was likely derived from a single queen mated to a single drone.

#### Woolacombe

A nest was discovered in Woolacombe on 27 September 2017. In total, 166 adult hornets were found in the nest, all female. The wet weight of adult female hornets ranged from 172 to 508 mg with a mean of 333 ± 5 mg (N = 166). Based on the information in Rome et al*.*^8^*,* described above, none of the females found in the Woolacombe nest were founder queens. The nest was 27 cm in diameter and is the largest nest discovered in England to date. The nest contained seven combs and all life stages were present, although the larval samples were too degraded for DNA analysis. Haploid individuals were present at the egg and pupal stages, the remainder of the individuals examined were diploid. Based on life stages present and the ploidy, the queen began laying haploid eggs on approximately 2nd September. The genetic analysis (results from COLONY2, verified manually) showed that the offspring sampled were likely to be the product of a single queen and three drones.

#### Fowey nests 1 + 2

Two nests were discovered in Fowey, Cornwall in 2018 on 3rd and 20th September, 40 m apart. The first nest contained three combs and had a diameter of 15 cm. No eggs or early instar larvae were present but late instar larvae, teneral adults and three adults were present. All individuals sampled were diploid. From the absence of eggs and early instar larvae, it was concluded that the queen was absent/missing in the 2–3 weeks prior to nest discovery. The second nest had a diameter of 19 cm and contained four combs with brood in all stages. Seven adult males and eight females were found in the nest; the males were diploid. All eggs and six of the ten larvae genotyped were haploid, while the pupae, teneral adults and adults were diploid. No queen was found. From the genetic analysis of the two nests, it was shown that both were highly likely to be offspring of a single queen and drone, with the first nest discovered presumably a primary nest and the other nest the secondary nest. From the ploidy of the life-stages present, it was inferred that the queen began laying haploid eggs around the 30th August.

#### New Alresford nest

The first nest found in Hampshire was discovered in New Alresford on 24th September 2018. The nest was 18 cm diameter and contained four combs with all life stages present. Twenty-eight males and 94 females were found. All the adults, teneral adults and pupae sampled were diploid, while within the larvae, five out of 10 were haploid, and within the eggs, two out of seven were haploid. The queen began laying haploid eggs around the 3rd September. The individuals from this nest were highly likely the offspring of a single queen and two drones.

#### Brockenhurst nest

The second nest found in Hampshire was discovered in Brockenhurst (approximately 30 miles away from New Alresford) and was destroyed on 04th October 2018. The nest was 18.5 cm diameter and there were three combs present with brood from the larvae stage onwards; no eggs were present indicating a recent loss of the queen or cessation of laying. All larvae and pupae were haploid and adult males were also haploid. The only diploid individuals present were worker females. The queen ceased laying before any diploid (future gyne) eggs were laid. The nest was consistent with being the offspring of a single queen mated with two drones.

#### Drayton Bassett nest

The first nest of 2019 was discovered on September 2nd at Drayton Bassett, Staffordshire. On arrival at Fera Science Ltd, the nest was too damaged to determine its size. Five adult female hornets were found in the nest. The wet weight of adult female hornets ranged from 197 to 312 mg with a mean of 271.6 (n = 5). The average wet weight of founder queens in September in the study by Rome et al*.*^8^ was 624 mg (n = 5). Based on this, it would appear that none of the females found in the nest were founder queens. All life stages were present in the nest, and all individuals genotyped were diploid. The nest was consistent with being the offspring of a single queen mated to a single drone.

#### Christchurch nests 1 + 2

On 01st October, 2019, a nest 13 cm diameter was discovered in Christchurch, Dorset. Two combs were present in the nest. One adult female hornet was found in the nest. The wet weight of this adult female hornet was 545 mg and the mesoscutum width was 4.6 mm. In a study by Pérez de Heredia et al.^16^ individuals taken from nests with a unimodal population had one individual per nest that had a mesoscutum width above 4.5 mm; no other individuals in these nests reached a mesoscutum width of 4.5 mm. It is therefore likely that the individual found in this nest was the queen. The combs contained eggs and larvae and had genotypes consistent with being the offspring of the queen that was present. Two eggs were haploid, the remainder of the eggs and larvae genotyped were diploid. On October 10th, a second nest was discovered in Christchurch, 10 m from the first nest, but could not be measured as it was intertwined with vegetation and fragmented upon removal. No adult hornets were found in the nest. Two combs were present, with capped and uncapped cells. Larvae, pupae and teneral adults were found, all of which were diploid. No eggs were found. Both nests from Christchurch were consistent with being the offspring of the same queen, mated to a single drone. The first nest found was likely to be the secondary nest, the second nest found likely to be the primary nest.

**Figure 2:**
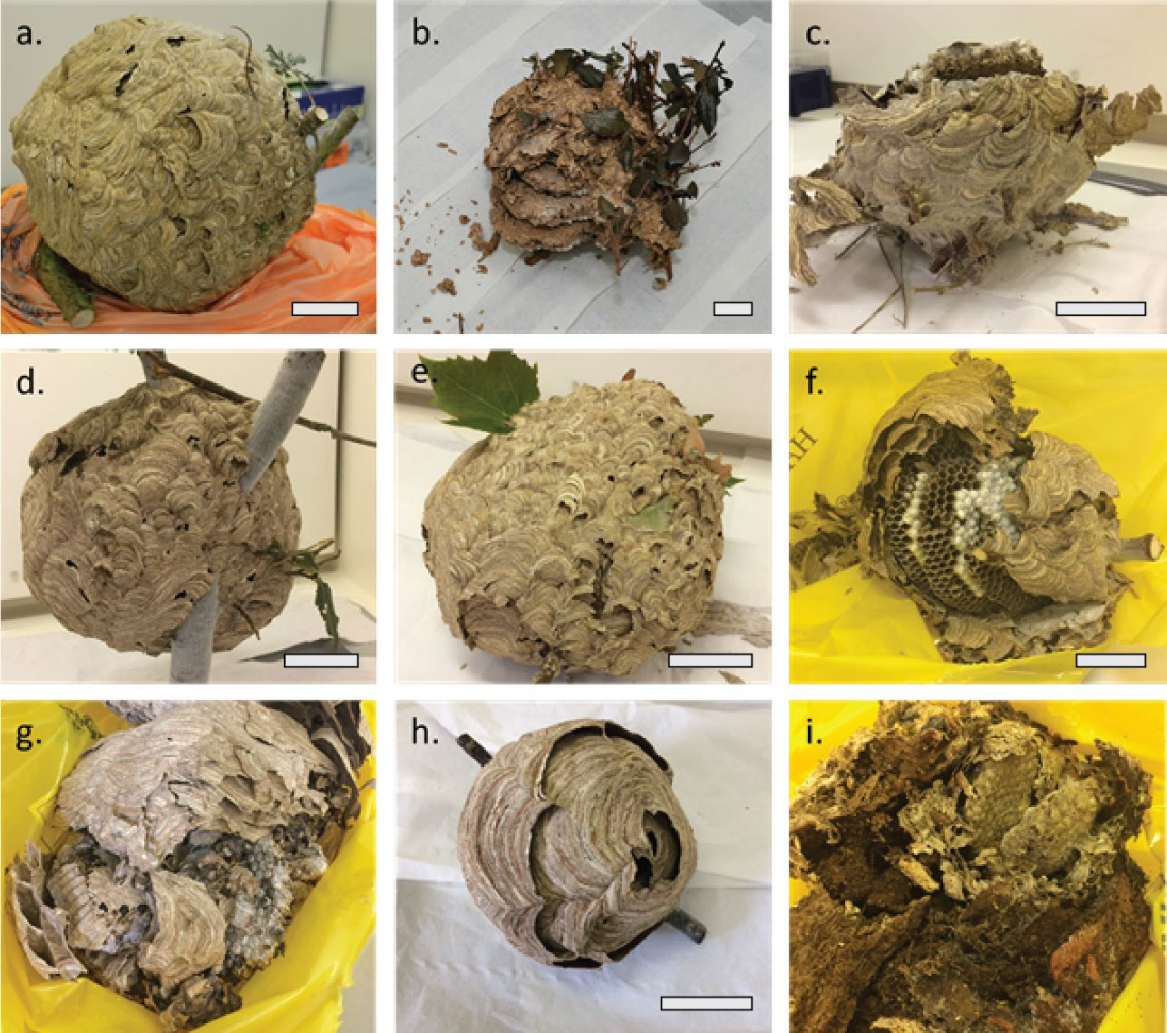
Images of UK nests: (**a**) Tetbury, (**b**) Woolacombe, (**c**) Fowey nest 1, (**d**) Fowey nest 2, (**e**) Brockenhurst, (**f**) New Alresford, (**g**) Drayton Bassett, (**h**) Christchurch nest 1 and (**i**) Christchurch nest 2. Where shown, scale bar represents 5 cm.

Information on all nests is found in Table 1. Map locations for each nest are shown in Fig. 1 and images of each nest are shown in Fig. 2.

### Genetic relatedness

Overall, the genetic diversity in the UK is relatively low for all locations, for all three measures used (mean number of alleles per locus, observed and expected heterozygosity; Table 3). However, it should be taken into consideration that the data for each UK nest are from individuals that were all closely related to each other (full, half siblings). A single combined figure for the UK was not calculated as it seems unlikely there is a UK population*.* Compared to the Asian hornet diversity data from Arca et al*.* (2015)^11^, the UK diversity is lower than France, which itself is lower than the diversity found in Asia (Table 2). This trend reflects the likely colonisation history of the hornet, which colonised France from Asia, and the UK incursions are likely to derive from populations on the European mainland.

**Table 3:** Genetic diversity measure (average number of alleles per locus) and the observed and expected heterozygosity for Asian hornet populations sampled in the British Isles, and from France (data from Arca et al. 2015^11^).

	Christchurch	Drayton Basset	Brockenhurst	New Alresford	Fowey	Woolacombe	Tetbury	France*	China (Yunnan)*	China (Zejiang)*
Number of samples	64	43	11	40	78	77	93	85	20	30
Observed heterozygosity	0.5299 *(0.3288)*	0.5081 *(0.4211)*	0.4430 *(0.3200)*	0.5017 *(0.3682)*	0.3962 *(0.3899)*	0.5714 *(0.3342)*	0.3816 *(0.3779)*	0.53	0.71	0.77
Expected heterozygosity	0.3391 *(0.1816)*	0.3120 *(0.2405)*	0.3277 *(0.2167)*	0.3259 *(0.2175)*	0.2630 *(0.2404)*	0.3886 *(0.1746)*	0.2422 *(0.2125)*	0.5542	0.79	0.79
Mean alleles per locus	1.80	1.80	1.80	1.87	1.73	2.00	1.60	4.00	7.3	8

Haploid individuals were excluded from the analysis. Figures and locations in italics are represented by single individuals.

*Data from Arca et al. 2015.

The occurrence of microsatellite alleles in the UK nests and France and Asia (from Arca et al.^11^) are given in supplementary material 1. In comparison with the Asian and French data in Arca et al.^11^, the UK samples had a restricted subset of alleles (35 alleles in total) that were all found in the French populations (60 alleles). In turn, all French alleles were a subset of those found in Asia (178 alleles). Similarly, the majority of private alleles were found in Asia (114), a small number in France (n = 3) and none in the UK (supplementary material 1).

In all cases, individuals recovered near a nest (within 2 km) were offspring of the recovered nearby nest (or nests, where there were primary and secondary nests). The majority of these individuals were found within 500 m of the nest. Most individuals caught in isolated locations away from nests (over 15 km) were not offspring of the recovered nests, with the exception of the individual recovered in Liskeard, which had a genotype compatible with being the offspring of the Fowey nest, some 17 km distant.

To exclude the possibility that any of the founding queens that escaped the destruction of the nest went on to produce viable nests that gave rise to nests caught in the subsequent year, we considered whether the inferred parental genotypes from a nest in year one could be the parents of the inferred parental genotypes in year two. For example, whether the queen from the Tetbury nest in 2016 could have formed a second nest and the offspring from that nest been the parents to the Woolacombe nest in 2017. In no case were the inferred parental genotypes compatible with this scenario. Additionally, where more than one nest was found in a single year (i.e. New Alresford/ Brockenhurst/ Fowey in 2018, Christchurch / Drayton Bassett in 2019), we considered whether the foundress queens and founder drones were full siblings to each other. Again, in no case was this possible (although they could have been half siblings to each other). Genotype data are provided in Supplementary material 2.

## Discussion

The origin of the hornets arriving into the UK have thus far been from continental Europe rather than Asia, as would be expected from the geographic proximity and density of transport links between continental Europe and the UK. This genetic relatedness is most obvious in the distribution of microsatellite alleles, where all the UK alleles (n = 35) are a subset of alleles found in France (n = 60), which in turn are a subset of alleles found in Asia (n = 178). The nests found do not appear to have directly given rise to each other, and a number of lone individuals are recorded each year, showing that multiple Asian hornets are arriving in the UK from mainland Europe each year. From the distribution of the nests in the UK, these are unlikely to be part of a natural dispersal, but to be individuals imported by the movement of people and goods^17^.

Nests discovered in the UK have been small (15–27 cm), the majority less than 20 cm, containing an average of fewer than four combs. The average hornet nest size in France during September is 7–10 combs^8^. The two largest UK nests found and destroyed in September had seven combs (Woolacombe) and five combs (Tetbury). Using data from the native and invaded range of *Vespa velutina nigrithorax*, it is predicted that the UK provides a suitable climate for the hornet^18^, while Keeling et al*.*^19^ suggest that the UK’s colder climate may affect the reproductive capacity of the hornet. The UK nests appear to be smaller than those found in France and this may be because the environment and climate in the UK are unsuitable for sustaining large, highly productive nests, or it could also be because the queens are founding their nests later in the season and therefore not reaching their maximum potential size.

*Vespa velutina nigrithorax* are polyandrous and queens in France were found to mate an average of two to four males^11^. In the UK nests, four out of seven queens were likely to have mated with a single drone, two queens mated with two drones and only one queen was mated with three drones. It is not possible to determine where the mating took place (e.g. mainland Europe or UK). A limited number of diverse mates gives rise to diploid male production (DMP). DMP is reported to occur in hornet nests in France due to severe inbreeding resulting in homozygosity at the sex locus in fertilised eggs^4^. Our results are consistent with this finding, with diploid males discovered in four out of seven UK nests (Tetbury, both Fowey nests and New Alresford), although, many nests had a limited number of adults to sample. Although DMP may limit colony growth due to the energetic dead-end of rearing diploid males, it does not appear to inhibit nests from entering the reproductive phase and it has not prevented this species from successfully colonising Europe. Only the Brockenhurst nest contained adult males that were haploid, however this nest contained haploids in all life stages which is suggestive of worker laid eggs (as has been recorded in *V. similima* and *V. affinis*^20^), or that the queen has exhausted the supply of sperm. Across Europe, *V.v. nigrithorax* is spreading and becoming established in new areas with an ‘invasion front’ spreading out across the continent. In other parts of Europe, after the initial discovery, increasing numbers of foraging hornets and nests are found in the same region in subsequent years: in north-west Italy, the hornet was regularly trapped in the two years after its first discovery in the region^21^, in Majorca, a single hornet nest was found in 2015 and nine more nests were discovered in the same region the following year^22^ and in Jersey sixteen nests were discovered the year following the first nest finding^23^. In contrast, in the UK, despite monitoring by the National Bee Unit and Asian Hornet Action Teams/AHATs at all locations after the initial nest discoveries, no foraging hornets or further nests were found, suggesting no subsequent or ‘hidden’ populations in these areas.

This is likely to be due to the relative geographic isolation of the UK, and to the management actions taken to control Asian hornets where they are found. After a confirmed sighting in the UK, all beekeepers registered with BeeBase, with apiaries within 20 km are alerted to the outbreak, are requested to monitor for hornets. Stakeholders and beekeeping associations are also informed. Local beekeeping associations have volunteer beekeepers, AHATs, who help members of the public identify and report sightings. During the search phase, apiaries within 5 km of the sighting are visited along with likely foraging sites and traps are deployed. When hornets are caught or seen visiting a site, track and trace techniques are used involving training marked hornets to bait stations and triangulating flight lines onto the likely location of the nest. Post destruction traps are monitored for the rest of the season and some traps are maintained into the following year during periods of likely flight activity.

Coupled with the fact that none of the nests discovered in the UK were directly descended from nests from previous years and that the inferred parental genotypes of nests discovered in the same year were not full siblings to each other, this is suggestive, but not conclusive, that there is no single established population occupying south England, but rather a series of incursions from mainland Europe. Keeling et al. (2017)^19^ use simulations to predict that a single nest could give rise to 16 nests by the third year and over 100 nests by the fifth year if uncontrolled in a region, eventually leading to high densities of nests in some regions leading to further pressure on honey bees and native pollinators. In the UK, the Non-Native Species Secretariat and the National Bee Unit respond to reports of foraging hornets, use trajectory tracking of foraging hornets to locate nests and destroy all nests found. Captured individual hornets and nests are sent back to Fera Science Ltd. and using microsatellite data, information can be gathered on the reproductive status of the nest helping inform the level of monitoring in the area for subsequent years, and whether individuals captured belong to one or many nests, thereby allowing inspectors on the ground to know how many nests they are searching for. This information feeds into real time management decisions and increases the chances for slowing the spread of the hornet. This policy so far has been successful in preventing uncontrolled expansion of hornets in the UK.

## Supplementary information

Supplementary Information 1.

Supplementary Information 2.

## Data Availability

All data generated or analysed during this study are included in this published article [and its supplementary information files].
